# Engineering Strategies to Overcome the Stability–Function Trade-Off in Proteins

**DOI:** 10.1021/acssynbio.1c00512

**Published:** 2022-03-08

**Authors:** Magdalena Teufl, Charlotte U. Zajc, Michael W. Traxlmayr

**Affiliations:** † Department of Chemistry, Institute of Biochemistry, University of Natural Resources and Life Sciences, 1190 Vienna, Austria; ‡ CD Laboratory for Next Generation CAR T Cells, 1190 Vienna, Austria

**Keywords:** protein stability, protein engineering, directed evolution, protein fitness, threshold robustness

## Abstract

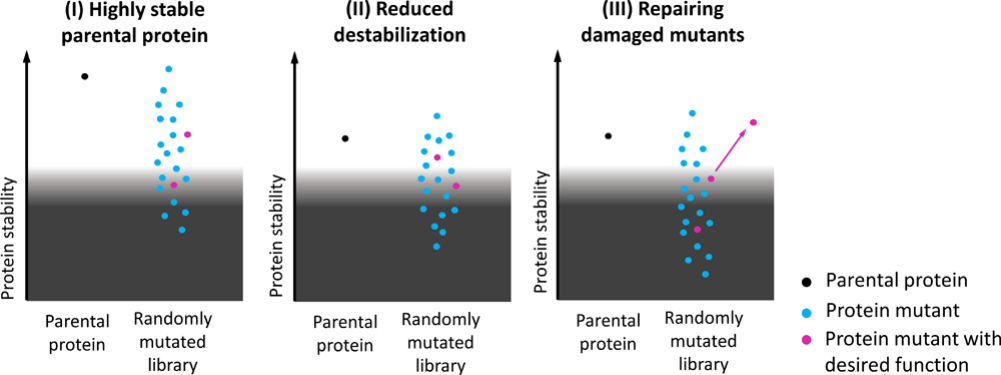

In addition to its biological function, the stability of a protein is a major determinant for its applicability. Unfortunately, engineering proteins for improved functionality usually results in destabilization of the protein. This so-called stability–function trade-off can be explained by the simple fact that the generation of a novel protein function—or the improvement of an existing one—necessitates the insertion of mutations, *i.e.*, deviations from the evolutionarily optimized wild-type sequence. In fact, it was demonstrated that gain-of-function mutations are not more destabilizing than other random mutations. The stability–function trade-off is a universal phenomenon during protein evolution that has been observed with completely different types of proteins, including enzymes, antibodies, and engineered binding scaffolds. In this review, we discuss three types of strategies that have been successfully deployed to overcome this omnipresent obstacle in protein engineering approaches: (i) using highly stable parental proteins, (ii) minimizing the extent of destabilization during functional engineering (by library optimization and/or coselection for stability and function), and (iii) repairing damaged mutants through stability engineering. The implementation of these strategies in protein engineering campaigns will facilitate the efficient generation of protein variants that are not only functional but also stable and therefore better-suited for subsequent applications.

## Practical Relevance of Protein Stability

Protein stability is a critical factor for the applicability of a protein. In many cases it is not sufficient if the protein can be expressed as a functional molecule. Instead, for a wide range of applications, high stability is demanded. Two prominent examples include enzymes used in industrial processes, which are required to be highly thermostable to allow elevated process temperatures,^1−6^ and therapeutic proteins, which need to maintain their native fold and function in human serum at 37 °C for at least several days or weeks.^7,8^

As a specific example, Willuda *et al.* demonstrated that a high-affinity single-chain variable fragment (scFv) directed against the tumor-associated antigen epithelial glycoprotein-2 failed to efficiently enrich in solid tumors in a mouse model.^8^ Enrichment was achieved only upon stabilization by grafting of its complementarity-determining regions (CDRs) into the highly stable 4D5 framework. This is a typical example of a protein that was functional (*i.e.*, it bound to its antigen with high affinity) but failed in its application because of insufficient stability.

In addition to the stability threshold dictated by the final application, protein stability is often correlated with the expression level.^9−13^ Several studies have reported that stabilization of poorly structured proteins increases expression yields by several fold,^7−12,14^ and stabilization of a single-chain TCR clone even improved its expression rate by more than 100-fold.^11^ Since a reasonable expression titer is required for practical applications, this well-documented correlation between protein stability and expression titer is a further important reason to aim for stable protein variants.

Another benefit of stable proteins is their tendency to show improved solubility.^11,15^ For example, stability engineering of a single-chain TCR clone by yeast surface display resulted in a 40-fold improvement of its solubility.^11^ A further important property related to solubility is protein aggregation. Although aggregation behavior and stability do not necessarily correlate in stably folded proteins,^16^ unstable proteins often tend to aggregate, possibly as a result of partial unfolding and exposure of hydrophobic residues.^8,15,17^ An additional or alternative explanation for this observation was proposed in a comprehensive *in silico* study on protein–protein complex structures by Pechmann *et al.*, who demonstrated that protein–protein interaction surfaces are more aggregation-prone than other protein surfaces. However, those “sticky” surface regions are frequently stabilized in their native states by disulfide bonds and salt bridges, thereby preventing nonspecific self-interactions (*i.e.*, aggregation).^18^ Thus, the increased aggregation tendency of unstable proteins may be explained by exposure of usually buried hydrophobic side chains due to partial unfolding and/or by increased flexibility of sticky surface residues.

## Parameters Describing Protein Stability

Despite the myriad conformations that would be possible even for a relatively short polypeptide, proteins possess the remarkable capability to reliably fold into their native structures. However, it is important to keep in mind that—even at room temperature—all native proteins are in equilibrium with their denatured states. This equilibrium between the native and denatured states is defined by the Gibbs free energy of unfolding (Δ*G*), which is a frequently used parameter to describe protein stability. That is, more stable proteins contain a smaller fraction of denatured molecules.

**Figure 1:**
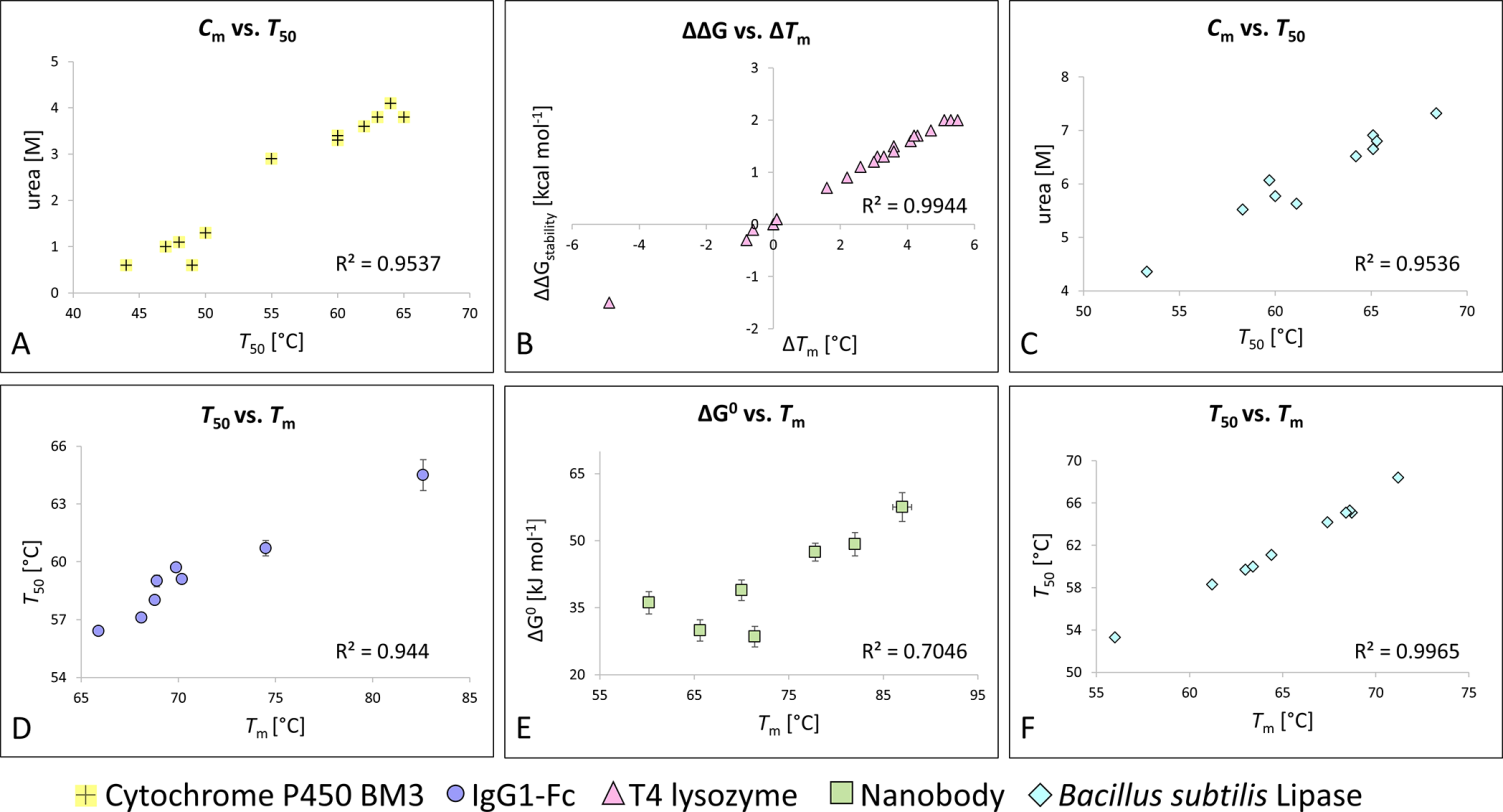
Correlations between different stability parameters. (A) Correlation between *C*_m_ and *T*_50_ for cytochrome P450 BM3 variants.^24^ (B) Correlation between ΔΔ*G* and Δ*T*_m_ for T4 lysozyme mutants.^25^ (C, F) Correlations between (C) *C*_m_ and *T*_50_ and (F) *T*_50_ and *T*_m_ for *Bacillus subtilis* lipase mutants.^1^ (D) Correlation between *T*_50_ and *T*_m_ for IgG1-Fc variants.^26^ (E) Correlation between Δ*G*° and *T*_m_ for disulfide variants of the nanobody cAbBCII10.^27^

Apart from Δ*G*, the thermal stability is also a frequently reported stability measure. The parameter most commonly used to describe the thermal stability is the midpoint of thermal denaturation (*T*_m_). Alternatively, the temperature at which 50% of the protein denatures irreversibly during a heat incubation step (*T*_50_) can be determined by analyzing protein activity (*e.g.*, enzyme activity or antigen binding) *after* the respective heat incubation.^19−21^ It should be noted that *T*_m_ and *T*_50_ are not identical but usually show very close correlations (Figure 1D,F).

Finally, also the resistance to denaturation induced by urea or other denaturing agents can be used to measure protein stability, *e.g.*, the concentration required to induce 50% denaturation (*C*_m_). It is important to keep in mind that Δ*G*, *T*_m_, *T*_50_, and *C*_m_ describe different aspects of protein stability. Nevertheless, in general there is a good correlation among those parameters, particularly when different mutants of the same protein are being compared, as shown in Figure 1. Therefore, for simplicity, in this review the term “protein stability” will be used as a general term describing the robustness of the native protein fold unless a specific stability parameter is indicated.

## The Stability–Function Trade-Off

Because of the pronounced effects of both biochemical function and folding stability on the biological activity of proteins, it is not surprising that both features have been extensively optimized by evolutionary processes. As a consequence, the majority of random mutations introduced into natural proteins or protein domains result in destabilization. In a comprehensive study by Tawfik and colleagues, the stability effects (ΔΔ*G*) of all possible mutations in 21 different globular single-domain proteins were calculated using the FoldX algorithm.^22^ Importantly, the authors also validated their *in silico* predictions with data from 1285 experimentally measured mutants. This study yielded several important findings: (i) most mutations in proteins are destabilizing, which is not surprising since the sequence deviates from its evolutionarily optimized version; (ii) the overall distributions of ΔΔ*G* effects of mutations are highly comparable between different proteins; and (iii) mutations of surface residues are on average considerably less destabilizing than those at core positions. Similar distributions of stability effects were observed in an experimental study in which the ΔΔ*G* values of almost all single mutants of protein G (Gβ1) were measured.^23^

Because of the destabilizing effect of most mutations, the introduction of a novel function into a protein or the optimization of an existing one is almost inevitably linked to a stability loss of the engineered polypeptide.^15,24,28,29^ That is, most mutations selected for gain of function are destabilizing. This phenomenon is often called the stability–function trade-off. Importantly, another study by Tawfik and colleagues demonstrated that the distribution of stability effects (ΔΔ*G*) of mutations that confer a new function is very similar to that of all possible mutations in the respective proteins.^30^ Those data indicate that the destabilizing effect associated with the acquisition of a novel function is primarily a consequence of the necessity to introduce mutations, but not because those mutations are particularly destabilizing. Exceptions are many key catalytic residues in enzymes, which are often highly destabilizing,^25,30^*e.g.*, because many of them are polar or charged but located in hydrophobic pockets.

Assuming that the mutational effects on stability are largely independent (*i.e.*, additive), one would expect that on average each mutation is equally likely to destabilize and thereby inactivate the protein. Therefore, it could be expected that protein fitness (*i.e.*, activity) declines exponentially with the number of inserted mutations. Indeed, such a relationship, which has been termed “gradient robustness”, has been observed for some loosely packed, marginally stable proteins such as those of highly mutating RNA viruses.^31,32^ However, for stable proteins the relationship between protein fitness and the number of mutations usually differs from the gradient robustness model since those proteins possess an extra margin of stability that can be exhausted before protein fitness declines considerably. That is, even though the first couple of mutations do compromise protein stability, they only marginally impair protein fitness. However, once the stability is reduced below a certain threshold, protein fitness declines rapidly.^24,28,32,33^ Therefore, this model is called “threshold robustness” or “negative epistasis”, the latter indicating that the negative effects of mutations on protein fitness are more than additive (because at some point the stability margin is exhausted and the negative effects become more apparent).

**Figure 2:**
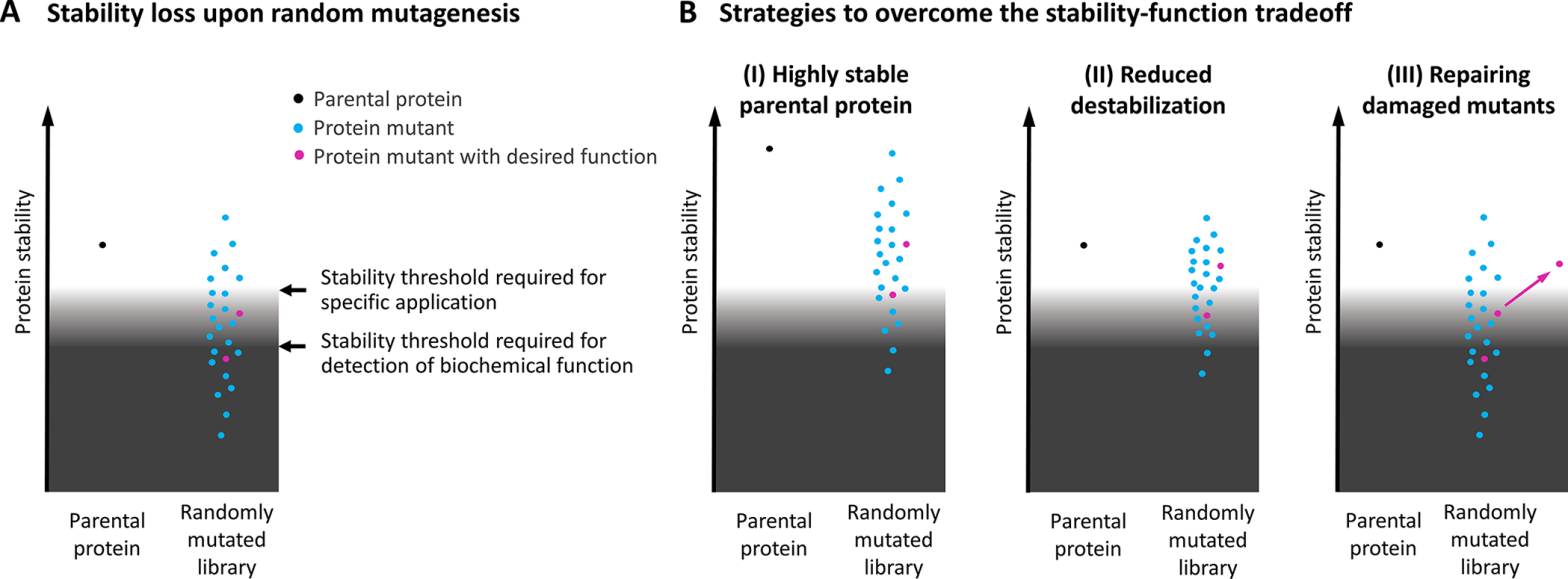
A simple model for the stability–function trade-off and strategies to overcome this obstacle. (A) The destabilizing effect of random mutagenesis is schematically depicted. It is assumed that several amino acid positions are randomly mutated simultaneously, *e.g.*, with the goal to engineer an artificial binding surface, resulting in destabilization of most mutants. (B) To overcome this stability–function trade-off, three main approaches have been described: (I) using highly stable parental proteins, (II) minimizing the destabilization associated with the inserted mutations, and (III) rescuing marginally stable proteins through various stabilization strategies.

Because of the considerable destabilizing effects usually observed upon generation of new protein functions, protein stability is a biophysical parameter that needs attention during any protein engineering process. Figure 2A depicts a simple model for the stability–function trade-off during protein engineering processes. In this model, it is assumed that several amino acid positions are randomly mutated simultaneously, *e.g.*, with the goal to generate a novel binding surface. As a consequence of this extensive mutagenesis, the vast majority of the resulting protein variants are destabilized. Furthermore, very few of those mostly destabilized variants show the desired function, *i.e.*, antigen binding (pink dots; Figure 2A). However, many of the polypeptides that would theoretically yield protein variants with suitable affinities do not fold into their native structures because of considerable destabilizing effects, precluding the detection of their biochemical function. Some other mutants are expressed as natively or partially folded proteins with antigen-binding activity, but they are too unstable for their intended application for various reasons discussed above. Thus, in this model system, two stability thresholds are defined: (i) a threshold that is required to yield a folded polypeptide with detectable function and (ii) a higher threshold dictated by the ultimate applications (Figure 2A).

In the model shown in Figure 2A, the protein mutants with desired antigen-binding capability (pink dots) are either misfolded or—if they fold into a functional protein—do not meet the stability threshold required for their final application. Since such a scenario is frequently observed in various types of protein engineering experiments,^8,15,24,34−36^ several strategies have been deployed to overcome this stability–function trade-off, which will be discussed in the following sections and are schematically summarized in Figure 2B.

## Strategy I: Highly Stable Parental Proteins

One approach is the use of highly stable parental proteins for the engineering process (Figure 2B, strategy I). This approach is based on the observation that stable proteins possess an extra stability margin that can be exhausted before their fitness is severely impacted, which is called “threshold robustness” as discussed above. In a landmark study with the telling title “Protein stability promotes evolvability”, Arnold and colleagues elegantly demonstrated that functionally improved variants could be evolved more efficiently from a thermostable cytochrome P450 BM3 heme domain (*T*_50_ = 62 °C) compared with its marginally stable counterpart (*T*_50_ = 47 °C).^24^ Starting the directed evolution experiments with the thermostable variant was shown to produce a wider range of functionally improved mutants compared with the unstable counterpart. Moreover, the thermostability of the obtained mutants was superior to that of the mutants evolved from the unstable P450 version. This study demonstrated that using highly stable proteins for protein engineering purposes confers two critical advantages: (i) improved evolvability due to increased mutational tolerance and (ii) more stable engineered variants (Figure 2B, strategy I *vs*Figure 2A). Importantly, the validity of the underlying threshold robustness model, *i.e.*, increased tolerance to mutation in more stable proteins, has been confirmed in further studies on other proteins and is therefore not an intrinsic property of the P450 heme domain but a general feature of proteins.^28,32,33,37^

**Figure 3:**
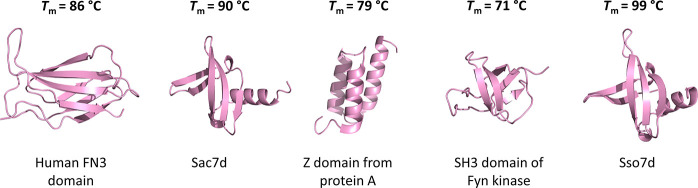
Comparison of the thermostabilities of five prominent parental binder scaffolds. The following structures are depicted: Sac7d (PDB ID 1AZP(52)); Sso7d (PDB ID 1BNZ(53)); SH3 domain of Fyn kinase (PDB ID 3UA6(54)); Z domain derived from Protein A (PDB ID 2SPZ(55)); FN3 domain (PDB ID 1FNF(56)). Depicted *T*_m_ values were extracted from refs (38), (39), and (46−48). The protein structures within this figure were generated using the PyMOL Molecular Graphics System.^57^

Given the stability-dictated evolvability of proteins, the use of hyperthermostable proteins as starting scaffolds for engineering approaches is an appealing strategy. For example, the small (7 kDa) hyperthermostable proteins Sac7d and Sso7d derived from the hyperthermophilic archaea *Sulfolobus acidocaldarius* and *Sulfolobus solfataricus*, respectively, have been used extensively for engineering of mini-binders with antibody-like affinities. Both proteins are extremely stable, with *T*_m_ values of 90 °C (Sac7d^38^) and 99 °C (Sso7d^39^) (Figure 3). As expected, these hyperthermostable proteins are highly tolerant of mutations,^39^ enabling the efficient generation of high-affinity binders against virtually any target molecule, including proteins, peptides, and small molecules.^38−45^

In general, most scaffold proteins used for binder engineering are highly stable and therefore relatively tolerant of mutations (Figure 3). Nevertheless, the *T*_m_ of Sso7d (99 °C) is markedly above those of other binding scaffolds. For comparison, the *T*_m_ of the SH3 domain of Fyn kinase (the wild-type domain of fynomers) is 71 °C,^46^ that of the Z domain derived from Protein A (the wild-type scaffold of affibodies) is 79 °C,^47^ and that of the 10th type III domain of human fibronectin (FN3 domain, the wild-type scaffold of monobodies) is 86 °C.^48^ Thus, although all of these other binding scaffolds are highly stable proteins, Sso7d provides a considerably higher stability margin that may be lost during the engineering process without any major impact on protein fitness. Moreover, apart from the improved evolvability provided by the extra stability, most binders derived from Sso7d are highly stable, with *T*_m_ values typically ranging between 60 and 100 °C,^39,41,45^ providing sufficient stability for most applications. Other examples of stable parental scaffolds efficiently used for protein engineering purposes include scFv libraries based on stable framework regions^49^ as well as designed ankyrin repeat proteins (DARPins), which were purposefully designed to be stable by consensus design.^50,51^

An alternative to the use of intrinsically hyperstable scaffold proteins is to improve the stability of the parental protein. Especially in cases where a particular biochemical and/or biological activity requires the choice of a relatively unstable protein as a starting scaffold, it is possible to initially increase the stability of the parental molecule prior to functional engineering.^24,36^ Strategies to improve protein stability include rational design^58−60^ and directed-evolution-based approaches,^13,21,61^ as will be further discussed below.

## Strategy II: Minimizing the Extent of Destabilization

### Improving Library Fitness

**Figure 4:**
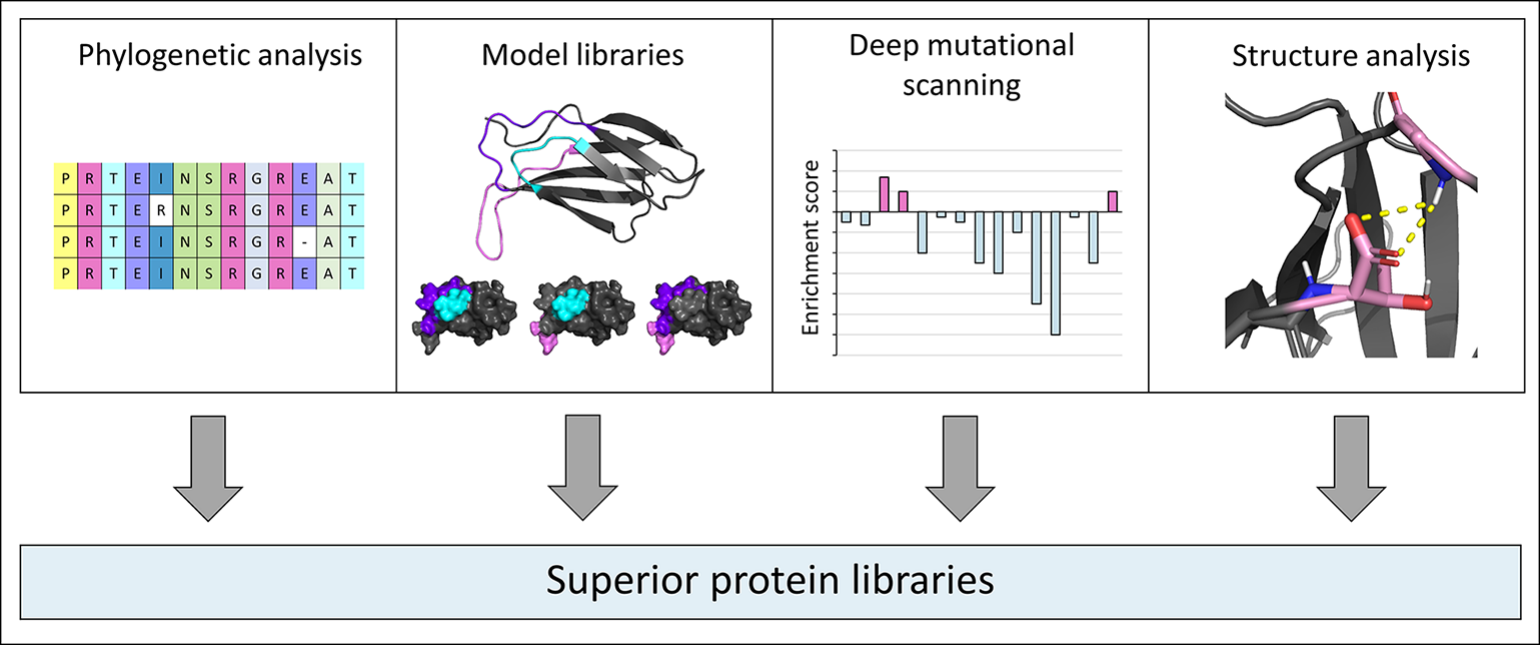
Strategies to reduce destabilization and to create superior protein libraries. Phylogenetic analyses reveal conserved positions, while model libraries (depicted here is PDB ID 1FNF(56)) can be used to identify mutation-tolerant regions. Additionally, deep mutational scanning and structural analysis (here PDB ID 1FNF is used as a schematic example) can provide further hints on the mutational tolerance of individual amino acid positions. The protein structures within this figure were generated using the PyMOL Molecular Graphics System.^57^

Apart from the stability of the parental protein, the stability loss associated with the engineering process is another important factor that should be considered (Figure 2B, strategy II). Several strategies have been reported with the overall goal to improve library fitness, *i.e.*, to minimize the destabilization caused by the insertion of random mutations (Figure 4).

As was discussed above, several comprehensive studies on the stability effects of mutations demonstrated that surface residues are considerably more tolerant to mutation than those buried in the protein core.^22,23,62^ As a consequence, it is highly advisable to focus the mutagenesis on solvent-exposed residues. While this might not (or might only partially) be an option for enzymes, which often possess relatively buried active sites, this basic rule is a good starting point in the design of libraries of antigen-binding scaffolds. In this case both the functional requirement to interact with antigens and the higher mutational tolerance call for mutagenesis at solvent-exposed positions. Thus, the availability of a high-resolution structure is a major advantage in library design. In addition to determination of the solvent accessibility, a structure also allows for inspection of side-chain interactions, which may also be a reason not to mutate a specific residue.

To further improve the quality of the library, the protein engineer should also—if possible—avoid mutagenesis at evolutionarily highly conserved positions since it is known that conservation in natural evolution is correlated with mutational intolerance.^62,63^ Thus, a phylogenetic analysis of the parental protein is a valuable resource for the choice of amino acid positions to be randomized in a library.^48^

Another powerful approach is the construction of model libraries, in which different positions or combinations of positions are randomly mutated followed by a high-throughput analysis of the resulting mutants, *e.g.*, by flow cytometry. For example, Hackel and colleagues generated a set of yeast-displayed FN3 libraries, including one with fully randomized loop regions, as well as several libraries that were identical to the fully diversified design except for wild-type conservation at a certain position (with each of those libraries showing wild-type conservation at a different loop position).^48^ High-throughput flow cytometric analysis of the resulting model libraries yielded stability factors (determined by full-length display on the yeast surface, which was shown to be correlated with the stability), indicating the contribution of wild-type conservation at each position within those loop regions. The information yielded from (i) those model libraries, (ii) a phylogenetic analysis of FN3 domains of various species, and (iii) the determination of the solvent-accessible surface area (SASA) of each candidate position enabled the construction of a next-generation FN3 library featuring full or partial wild-type conservation at selected positions. This improved library (termed fourth-generation, G4) performed significantly better than control FN3 libraries when analyzed by flow cytometry. Moreover, to directly compare its capacity to yield functional antigen-binding variants, the improved G4 library was pooled with two similarly diverse control libraries followed by selection for binding to seven different targets. Remarkably, sequence analysis of the enriched binders demonstrated that 19 out of 21 mutants were derived from the G4 library, clearly demonstrating the superiority of this library design that was based on experimental results with model libraries as well as structural and phylogenetic analysis.^48^

Apart from model libraries with wild-type conservation at selected positions such as those described above, also other model library designs have proven to be informative for the generation of high-quality libraries. Hasenhindl *et al.* constructed a set of yeast display libraries with full randomization at different amino acid stretches within structural loop regions of the C_H_3 domains of human IgG1-Fc.^26^ Subjecting the yeast display libraries to different heat incubation temperatures and subsequent analysis of binding to structurally specific ligands enabled the determination of the overall thermal stabilities (*T*_50_) of the different library pools. These experiments revealed pronounced differences in the mutational tolerance of different loop segments. For example, randomization of only two positions (R416 and W417) within the EF loop led to slightly stronger destabilization than full diversification of a much larger fragment spanning the five neighboring residues (418–422). The pronounced mutational intolerance of residues R416 and W417 may be explained by a salt bridge formed between R416 and E388 and the positioning of the side chain of W417 in the hydrophobic core of the C_H_3 domain.

Deep mutational scanning is an alternative experimental approach to determine the mutational tolerance of different amino acid positions. Briefly, this method is based on high-throughput selection of a randomly mutated library followed by deep sequencing analysis and determination of enrichment scores for each mutation in the library.^63^ Deep mutational scanning of IgG1-Fc yielded a stability landscape of the entire C_H_3 domain at single-residue resolution.^62^ That is, the mutational tolerance of each amino acid position could be determined within a single experiment. Despite the differences in the experimental approaches, the mutational tolerances in this stability landscape were highly consistent with those obtained from the model libraries in the C_H_3 domains.^26,62^

### Coselection for Stability and Function

**Figure 5:**
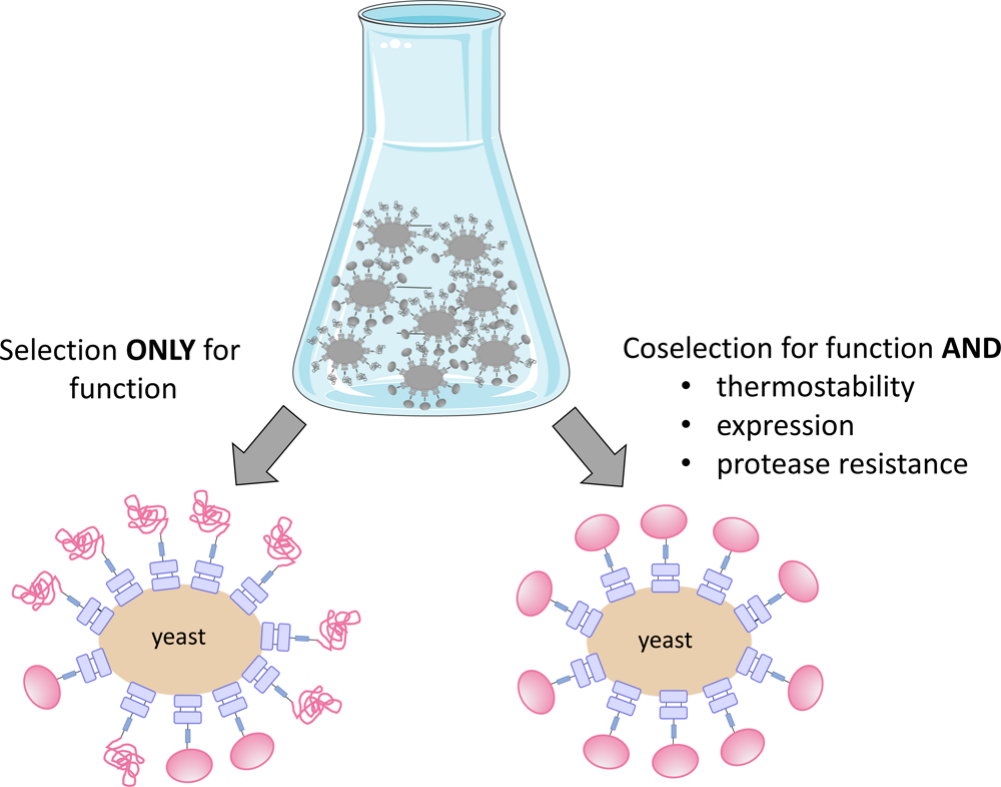
Coselection for function and stability. Selection for function only may yield highly affine (or enzymatically active) but destabilized mutants. Thus, coselection for further parameters such as thermostability, expression, and protease resistance can be used to yield variants that are both functional and stable. Part of the image was prepared by adaptation of content from Servier Medical Art (https://smart.servier.com/).

Besides choosing optimal positions for randomization, a further approach to minimize destabilization during directed evolution is coselection for both stability and function (Figure 5). In two elegant studies by the Tessier lab, the evolutionary mechanisms in yeast display-guided affinity maturations of human single domain (V_H_) antibodies were studied. In initial experiments, the selection pressure was directed primarily toward improved affinity, yielding affinity-matured but strongly destabilized V_H_ domains, thus representing a classic example of a pronounced stability–function trade-off.^34^ To prevent this considerable stability loss, the authors performed additional directed evolution experiments in which they coselected for both affinity and stability. This improved selection strategy enabled the enrichment of affinity-matured variants that were only slightly destabilized.^34^ As expected, detailed analysis of the mutations accumulated during coselection for stability and affinity revealed that several affinity-enhancing mutations showed destabilizing effects. Interestingly, two stabilizing mutations partially compensated for this destabilization, improving not only the stability but also the affinity.^35^ Together, these two studies nicely demonstrate that coselection for improved function and stability is possible and that a protein can adapt to this dual selection pressure by accumulating a set of mutations with positive effects on protein function and/or stability.

It should be noted that this phenomenon has been observed not only in the laboratory but also during natural evolution. For example, in the course of evolutionary adaptation of β-lactamases in response to novel antibiotics, function-enhancing mutations are often destabilizing and therefore require coenrichment of stabilizing mutations to compensate for the stability loss.^64,65^

Affinity maturation of antibodies in B cells represents another example of a natural evolutionary process where coselection for function and stability has been observed. In a high-throughput study by Shehata and colleagues, hundreds of antibodies derived from different human B cell compartments were analyzed with respect to their content of somatic mutations acquired during affinity maturation *in vivo* as well as their thermal stabilities.^66^ mAbs derived from naïve B cells showed significantly higher thermal stabilities than those derived from B cell populations that had undergone affinity maturation. The authors also provided additional experimental evidence that the introduction of somatic mutations during affinity maturation was indeed responsible for the destabilization, thus representing a perfect example of a stability–function trade-off. Importantly, while the first 10 somatic mutations led to a statistically significant decrease in the thermal stability, there was no further destabilization in mAbs containing 11–20 or even more than 20 mutations.^66^ This strongly suggests that there is a stability threshold that needs to be maintained to be competitive in the germinal center *in vivo*, thus demanding coselection for stability and function once this stability threshold is reached.

Likewise, natural evolution of proteins is currently being experienced by humankind in real time in the course of the SARS-CoV-2 pandemic. Various mutations have emerged over time, and some—among them the D614G mutation in the spike protein (S)—have been shown to persist,^67−70^ indicating a survival advantage over other variants. Indeed, increased infectivity of the D614G variant was shown in several cell culture experiments^67,70,71^ and seems to be at least partially mediated by a shift of the spike protein conformation toward a receptor-binding and fusion-competent state.^70^ Interestingly, several research groups computationally predicted a stabilizing effect of D614G on the spike protein structure, which may further confer a selective advantage.^68,69,72^ Furthermore, Teng *et al.* computationally analyzed the stability effects (ΔΔ*G*) of all possible S protein mutations.^69^ Interestingly, comparing the set of theoretically possible mutations to 237 viral missense variations that had been reported to that date, they found (i) a distinct over-representation of stabilizing mutations and (ii) a pronounced depletion of strongly destabilizing mutations in the cohort of naturally occurring viral variants. Similarly, in another study, an *in silico* analysis of mutations enriched in circulating SARS-CoV-2 strains revealed a remarkable balance between stabilizing and destabilizing mutations in various SARS-CoV-2 proteins.^72^ Together, these studies strongly suggest that there exists a selection pressure toward maintained (or even improved) stability of SARS-CoV-2 proteins, thus representing an illustrative real-life example of coselection of protein stability and function.

## Strategy III: Repairing Damaged Mutants

In cases where functional improvements cause severe destabilizing effects that preclude application of the engineered protein, it is possible to repair the mutants through stabilization (Figure 2B, strategy III). Of course, maintenance of sufficient stability is a prerequisite to be able to enrich the lead candidate from a library based on its biochemical function. Thus, repairing “damaged” variants should only be considered an option for unintentionally strongly destabilized mutants, but it should not be part of a standard protein engineering pipeline. Instead, the other two strategies described above, *i.e.*, use of highly stable parental proteins and/or minimization of the stability loss during functional engineering, should be preferred because those approaches not only yield more stable engineered variants but also increase the functional diversity of the original library and thereby the evolvability of the protein.

**Figure 6:**
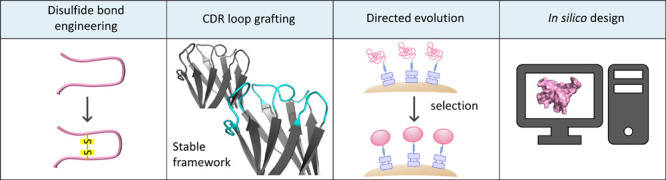
Strategies to repair damaged protein mutants. Selected examples of protein stabilization strategies include the introduction of additional disulfide bonds, CDR loop grafting onto stable framework regions (here the stable framework 4D5 (PBD ID 1FVC(78)) is used schematically for illustration purposes), yeast display selections, and computational design (PDB ID 1AZP(52) is depicted as a representative example). The protein structures within this figure were generated using the PyMOL Molecular Graphics System.^57^

Nevertheless, in certain cases it is worth repairing promising lead candidates suffering from low stability (Figure 6). For example, the engineered Her2-binding Fc antigen binding (Fcab) clone H10-03-6 showed promising biological activity against Her2-positive cancer cell lines both *in vitro* and *in vivo*,^73,74^ but it suffered from relatively low stability and its tendency to aggregate. Yeast display-based selection for maintained binding to the antigen and to a structurally specific ligand after a heat incubation step yielded a variant with slightly adapted antigen-binding-loop regions. This mutant showed increased thermal stability, strongly improved resistance to aggregation, and increased solubility, albeit with a slight (∼5-fold) loss in affinity.^15^ In alternative approaches, the same Fcab lead candidate H10-03-6 was stabilized by the introduction of non-native disulfide bonds into its C_H_3 domains,^59,60^ demonstrating that different strategies can be applied to rescue destabilized engineered mutants. An increase in stability upon rational engineering of disulfide bonds was also shown for various other proteins, including nanobodies,^27,75^ domain III of *Pseudomonas* exotoxin A,^76^ and a thermolysin-like protease,^77^ validating this strategy as a generally applicable approach for protein stabilization.

Grafting of antibody CDR loops onto stable frameworks is another stabilization strategy that is particularly attractive if the lead candidate is a hybridoma-derived non-human antibody, since in those cases the stabilization may be combined with a humanization process (if a human acceptor framework is chosen). McConnell *et al.* generated a highly stable antibody framework by combining several stabilization strategies, including the choice of stable human framework regions, consensus design, introduction of additional disulfide bonds, and computational design.^79^ Remarkably, engraftment of CDR regions derived from 10 different human or murine antibodies onto this optimized antibody framework resulted in stabilization of eight out of 10 mAbs.^80^ Moreover, in all cases but one, the affinity was maintained within 3-fold compared to the parental antibodies, which is remarkable since CDR grafting is usually associated with significant affinity loss.^81,82^ Thus, besides antibody humanization, CDR grafting can generally be applied to improve the thermal or chemical stability of antibodies for various applications.^83,84^

CDR grafting onto stable protein scaffolds has also been successfully performed with scFvs.^8,85^ Moreover, even the antigen-binding loops of an engineered FN3 domain have been successfully grafted onto a stable FN3 scaffold obtained by consensus design,^17^ suggesting that stabilization achieved through loop grafting is more generally applicable and not limited to antibody CDRs.

## Conclusion

Overall, it can be concluded that protein stability plays a critical role during protein evolution both in the laboratory and in nature. Implementing approaches that aim for higher stability in the protein engineering process from the beginning not only yields protein variants with superior characteristics but also reduces subsequent time- and work-intensive efforts to repair unstable proteins. Thus, the frequently observed stability–function trade-off should be circumvented with a range of approaches as discussed above, which can be combined to achieve not only functional but also highly stable and readily applicable proteins.
